# Understanding the log file data from educational and psychological computer-based testing: A scoping review protocol

**DOI:** 10.1371/journal.pone.0304109

**Published:** 2024-05-23

**Authors:** Guanyu Chen, Yan Liu, Yue Mao

**Affiliations:** 1 Department of Educational and Counselling Psychology, and Special Education, The University of British Columbia, Vancouver, Canada; 2 Department of Psychology, Carleton University, Ottawa, Canada; Zhejiang Normal University, CHINA

## Abstract

With the advancement of computer-based testing, log file data has drawn considerable attention from researchers. Although emerging studies have begun to explore log file data, there is a gap in the exploitation of log file data for capturing and understanding participants’ cognitive processes. The debate on how to maximize insights from log file data has not yet reached a consensus. Therefore, we present this protocol for a scoping review that aims to characterize the application of log file data in current publications, including the data pre-processing techniques, analytical methodologies, and theoretical frameworks used by researchers. This review will also aim to illuminate how log file data can enhance psychological and educational assessments. Our findings will highlight the opportunities and challenges presented by log file data as an emerging and essential source of evidence for future advancements in psychological and educational assessment.

## Introduction

With the recent development of computer technology, the collection of log file data in computer-based testing (CBT) has become a common practice [1]. Log file data, generated as a by-product of data collection in CBT [2], provide a detailed record of the test takers’ interactions with the computer when they understand, evaluate, and generate answers to assessment tasks [3]. More than just the final answers, log file data contains a wealth of information that reflects the underlying cognitive activities of test takers in CBT.

The application of log file data offers promising advancements in the field of psychological and educational measurement. Specifically, log file data records the response processes of test takers, offering insights into their test performance, behavior patterns, and cognitive strategies [4]. This rich information goes beyond simple score outcomes, enabling a more detailed interpretation of test-taker proficiency. Integrating log file data opens a new avenue for evaluating the validity of assessments [5]. Moreover, log file data provides an in-depth view of group difference that can reveal potential causes of test fairness. With their abundant potential for application, log file data is set to emerge as a valuable resource for educators, policymakers, and test developers.

While the benefits of log file data are significant, they also present challenges. These challenges were anticipated by Luecht and Clauser [6], who noted, “the real challenge is how to filter, encode, smooth and raw score the data to retain as much information as necessary.” In practice, the complexity of log file data raises questions about which test takers’ actions should be recorded and how to derive meaningful inferences from them [1]. As a result, researchers lack a consensus on these processes of recording, encoding, transforming, and modeling log file data.

Unfortunately, there are no systematic reviews addressing these complexities, and we plan to conduct a scoping review that will help researchers examine the use of log file data, classify analytical methods, summarize the key findings, and identify existing gaps [7]. Developing a protocol of such scoping review, including the theoretical and analytical framework of log file data, criteria for selecting literature, the procedure of database searches, and expected outcomes, will lead to a comprehensive understanding of the utilization of log file data from CBT in education and psychology.

### Log file data

Log file data can be traced back to the log files that record events occurring in a computer system [8, 9]. Log file data documents the interactions between the test takers and the computer [10]. In CBT, both the test takers’ actions to the stimulus materials (i.e., events) and the temporal sequence of these actions (i.e., the timestamp) are stored in log file data [3, 4, 11].

For illustration purposes, Table 1 shows an example of log file data. Typically, this data is usually stored in a structured format, such as Extensible Markup Language (XML) and JSON (JavaScript Object Notation), and need to be parsed and converted into a tabular data frame for further analysis [12]. This example is from a problem-solving task in Programme for International Student Assessment (PISA) 2012. The first three columns denote the country, school ID, and student ID. The fourth column contains the type of event, including system-generated events (start item, end item) and student-generated events (e.g., ACER_EVENT, click). Note that ACER_EVENT represented the manipulation of interactive elements (i.e., a Boolean vector that describes the state of highlighted roads segment on the map) in the problem-solving task [13].The fifth column records the timestamp, given in seconds from the beginning of the assessment. The sixth column is the event sequence number. The seventh column provided detailed information (i.e., properties) about the event.

**Table 1:** Example of log file data from PISA 2012 problem solving.

cnt	schoolid	StIDStd	event	time	event_number	event_value
ARE	0000189	04855	START_ITEM	0.10	1	NULL
ARE	0000189	04855	ACER_EVENT	43.40	2	’00000000000010000000000
ARE	0000189	04855	click	43.40	3	hit_nowhereSakharov
ARE	0000189	04855	ACER_EVENT	44.90	4	’00000000000000000000000
ARE	0000189	04855	click	44.90	5	hit_nowhereSakharov
ARE	0000189	04855	click	47.20	6	map
ARE	0000189	04855	click	50.10	7	timeMinutes
ARE	0000189	04855	click	53.00	8	timeMinutes

This data was downloaded from https://www.oecd.org/pisa/pisaproducts/database-cbapisa2012.htm.

### Opportunities and challenges on log file data

In the fields of education and psychology, endeavors have explored the application of log file data. Current research has highlighted the four main contributions: understanding the behavioral and cognitive strategies of test-takers, providing validity evidence of CBT, providing insights on test fairness, and assessing students’ performance. These insights collectively underscore the significant impact of log file data in advancing testing methodologies and practices.

First, understanding the behavioral and cognitive strategies of test-takers is an important topic in educational and psychological assessment. The interpretation of test scores carries assumptions about the cognitive processes that test takers employ [5], and log file data illuminates these processes during assessment. For example, Ren et al. [14] utilized log file data to discern three different goal pursuit strategies (demand, price, and availability) for the “Ticket” task in the PISA 2012 problem-solving test, and a significant relationship was found between the goal pursuit strategies and students’ performance. In another study, Jiang et al. [15] pinpointed varied response strategies, such as source-focused, target-focused, mixed, and indistinguishable, in drag-and-drop tasks of a mathematics assessment. A significant difference in performance was observed across these strategies. Adopting log file data provides deeper insights into how cognitive strategies impact test results.

Second, log file data represents an emerging form of validity evidence. The Standards for Educational and Psychological Testing emphasized the importance of response processes as a form of validity evidence for the interpretation of test scores [5]. For instance, a study by the OECD [10] revealed three timing indicators (e.g., time to first interaction, time since the last action, and the total time on a task), had significant associations with both the proportion of unattempted questions and overall performance. Beyond temporal indicators, Stadler et al. [16] identified typical patterns of actions leading to successful performance, thereby highlighting differences between successful and unsuccessful test-takers. Similarly, Qiao and Jiao [17] transformed log file data into action-based predictors, identifying key actions as determinants of predicting students’ performance. Hence, log file data provides a promising avenue for understanding test-takers’ performance and interpreting test scores.

Third, log file data offers valuable insights into test fairness, particularly regarding the consistent interpretation of test scores across diverse subgroups of test-takers. Research has found distinct behavioral patterns across demographics such as education level, income, gender, age, and familiarity with Information and Communication Technology (ICT) during problem-solving tasks [18, 19], exploring potential factors behind subgroup differences. Furthermore, log file data facilitates a detailed exploration of such differences. For example, Guo et al. [20] found that individuals from lower socio-economic status (SES) groups spent more time on text production and editing to generate essays of comparable quality, resulting in lower efficiency and typing speed. In sum, ensuring fairness is crucial for educational and psychological assessment, and log file data deepens our comprehension of differences among subgroups.

Finally, log file data has demonstrated potential in evaluating student performance. Weber et al. [21] have incorporated student behavior as additional indicators for scoring in s inductive reasoning tests, showing an advantage over traditional scoring methods that rely solely on the sum of correct items for predicting grade point average. Furthermore, Bergner and von Davier [22] suggested using scores derived from log file data as outcomes, rather than treating log file data as auxiliary to outcomes. For instance, Scoular et al. [23] and Drake et al. [24] have demonstrated how student behavior from log file data, can be scored to assess performance in collaborative problem-solving tasks. And various models have been developed to estimate problem-solving abilities from action sequences [25, 26] and student speed from action time [27].

While log file data offers promising opportunities, it also poses challenges for researchers in its recording and utilization. A primary challenge lies in determining the optimal information to record. This logging decision must consider and select the suitable test-takers’ interactions that can provide actionable and interpretable evidence about test-takers’ proficiency, understanding, or strategies [28]. In practice, the logging choices have mainly been determined by the software engineers of CBT, resulting in the omission of some potentially important actions [10]. Therefore, researchers can consider using the evidence-centered design as a comprehensive framework to identify significant actions for recording [12], and work closely with the software engineers to develop a theory-based logging system.

Further complicating matters is the challenge of drawing meaningful inferences from log file data. To begin with, researchers may encounter the varied logging events in practice, not limited to click actions as illustrated earlier, but also encompassing copy, cut, paste, drop, keypress, and so on [29]. Furthermore, the raw data is typically stored in XML or JSON formats, diverging from the conventional data structures in psychology and education [10]. Adding to the complexity, log file data involves a huge amount of raw actions, and there is no straightforward way to generate a variable that captures a meaningful cognitive process [30]. Moreover, while log file data records the test takers’ actions, the underlying cognitive mechanisms are not clear. In sum, the current research lacks a universally accepted analytical method and theoretical framework to bridge the gap between the raw log file data and meaningful interpretation of cognitive abilities.

### Why conduct a scoping review on log file data

Although log file data is an emerging topic and several empirical studies have been conducted, no review has been carried out to offer insights into the current applications related to log file data according to our best knowledge. A scoping review can be conducted as a stand-alone study to map the key concepts behind a research topic and different sources of evidence, especially for a complex and emerging topic [31]. Conducting such a scoping review will contribute to an overall understanding of the current application of log file data across different research topics in educational and psychological assessment.

Specifically, we will undertake the scoping reviews to examine the extent and characteristics of research involving log file data. Such an endeavor is crucial for understanding the application and analysis of log file data. By summarizing the characteristics of current research, this scoping review will elucidate both the analytical and theoretical frameworks applied in log file data studies, offering a comprehensive perspective on the potential opportunities, challenges. Finally, the insights gained from this review will not only inform future research directions but also influence practical methodologies in the field.

### Review objects

This scoping review aims to systematically investigate how log file data is used in CBT, especially in the field of psychology and education to answer the following questions:

Q1: What are the characteristics of the studies using log file data from CBT?
Q2: What specific research objectives and purposes have guided the application of log file data?
Q3: Given the complexity of log file data, what kind of methods have been employed to analyze log file data?
Q4: Which theoretical frameworks have been applied to the analysis of log file data?
Q5: What are the major opportunities and challenges associated with the use of log file data?

## Methods

### Study design

We adopted the six-stage framework to organize this scoping review [31–33], including (1) identifying the research question, (2) identifying relevant studies, (3) study selection, (4) charting the data, (5) collating, summarizing and reporting the results, and (6) consultation exercise. Given that the first stage has been mentioned in the Review Objects section, the next five stages will be described below consistent with the objectives of the current scoping review. Additionally, we adheres to the guidelines set forth by the Preferred Reporting Items for Systematic Reviews and Meta-Analyses Extension for Scoping Reviews (PRISMA-ScR) [7, 34], and we have considered the items from PRISMA-ScR to design our scoping review protocol (see S1 Checklist).

### Search strategy

According to the six-stage framework, we need to consider how to identify the relevant studies as comprehensively as possible [31]. Peters et al. [35] recommended a three-step search strategy for developing a scoping review, and this strategy will be used. The first step is an initial limited search of Web of Science, and the analysis of the keywords contained in the title and abstract of retrieved papers from the initial search will be conducted. Then, the second search will use and update the keywords for all the included databases, including ERIC, Education Source, PsycInfo, ProQuest Dissertations & Theses Global, Web of Science, and Scopus. Thirdly, we will also check the reference list of identified articles as supplement sources.

Informed by the Peer Review of Electronic Search Strategies (PRESS) checklist [36, 37], we delineated two key search concepts from our research questions: log file data and CBT. To ensure our discussion focuses on log file data within the context of CBT, we employed the Boolean operator “and” to combine these concepts. Subsequent text word searches addressed various spelling variants and terminologies related to log file data and CBT. Additionally, our search encompassed several prominent large-scale computer-based assessments, including Programme for International Student Assessment (PISA), National Assessment of Educational Progress (NAEP), Programme for the International Assessment of Adult Competencies (PIAAC), Progress in International Reading Literacy Study (PIRLS), and Trends in International Mathematics and Science Study (TIMSS). These assessments represent major open sources of log file data and are integral to our research scope.

The refined search query, detailed in S1 File, comprises two components: (1) terms associated with log file data, including “process data”, “log data”, and “log file”; (2) terms pertinent to computer-based testing, such as “computer based test”, “assessment”, “scenario based”, and so on. This query returns 1546 records in Web of Science and 2146 records without duplicates from all databases. We designed this query to cover the relevant concepts of log file data and the scope of literature on CBT in the field of education and psychology.

### Inclusion and exclusion criteria

In this scoping review, we consider all types of empirical research, including gray literature from ProQuest Dissertations & Theses Global. Note that we specifically focused on the theoretical and analytical frameworks of log file data in practice. We will also consider methodological studies if the paper includes case studies or data demonstration. Studies that only focus on theory, simulation, or algorithm will be excluded in this review. Additionally, we will exclude all types of review studies, such as, systematic review, meta-analysis, and narrative review. We will include peer-reviewed journal articles, books, and book chapters, published from 2000 to 2023. Moreover, we are limited to including only full-text articles in English for conducting the full-text review, in accordance with the reviewers’ language capabilities. Finally, there is no restriction on human populations involved in the studies.

### Study selection

Study selection is the third stage of organizing a scoping review [31]. This is an iterative, rather than linear, stage involving a process of searching the literature, refining the search strategy, and reviewing articles for study inclusion and exclusion criteria [33].

At least two independent reviewers were asked to perform the study selection for title and abstract screening and full-text screening. Another content expert was invited to solve disagreements between the reviewers. Note that the selection is carried out by pre-specified inclusion and exclusion criteria from this review protocol. Some pilot testing was recommended prior to the formal selection for refining this study selection process [35]. We have chosen a sample of 50 articles, review these articles with eligibility criteria, discuss the discrepancies, and modify the search query and eligibility criteria.

We will use a flowchart of the review process from PRISMA-ScR to describe the whole scoping review process, including the databases, duplications, screening, full-text retrieval, and additional search from reference lists and relevant organizations. Covidence will be used for data management and screening.

### Data extraction

The fourth stage involved ‘charting’ information from the included studies [31]. Arksey and O’Malley liken charting to a “narrative review” process, wherein details from each article are meticulously recorded. This aids in contextualizing findings and making comparisons across studies. To ensure a broad and adaptable framework for our subsequent scoping review of log file data, it is vital to extract data that embodies the characteristics of log file data applications.

Drawing inspiration from other scoping reviews that focus on the application of advanced methods [38–40], we will record a series of key information, such as:

1. Bibliometric information: authors, publication year, and journal
2. Theoretical framework: research domain, cognitive variables, and theories
3. Analytical framework: data source, data cleaning process, analytical methods, performance metrics, and cross-validation
4. Implications: study purposes, findings, and any opportunities and challenges due to the use of log-file data

We will employ Google Forms to design a data charting form tailored to gather this specific information, aiding in addressing our research questions. Note that as the review progresses, additional data may be incorporated, necessitating regular updates to the chart form. To enhance the reliability and accuracy of our data extraction, once the review team finalizes the charting form through discussions and trials, two independent reviewers will undertake the extraction process to ensure the quality of data extraction. Due to most of research for the log file data belongs to observational study, we consider to use STrengthening the Reporting of OBservational studies in Epidemiology (STROBE) for quality assessment [41].

### Data synthesis

The subsequent phase involves collating, summarizing, and reporting our findings [31]. For clarity, we will segment this phase into three distinct steps, following the guidance of Levac et al. [33]: data analysis, results reporting, and implications discussion.

For data analysis, our scoping review employs several strategies. Firstly, we utilize a bibliometric analysis, leveraging the R package, *bibliometrix*, to highlight the primary trends associated with log file data [42]. Secondly, through taxonomical classification, we strive to identify and classify widely used cognitive framework for log file data. Thirdly, our summary analysis delves into the characteristics of log file data applications, emphasizing key dimensions like the research domain, data source, and method [31, 35]. Given the focus on machine learning approaches in log file data, we aspire to scrutinize the performance of these machine learning models, especially focusing on the role of cross-validation in assessing the generalizability of studies. Fourthly, thematic analysis seeks to elucidate the opportunities and challenges associated with different methods, providing insights for future researchers [43].

In terms of Results Reporting, each analysis type corresponds to a specific output. (1) A trend plot will visualize the progression of log file data publications [44]. (2) A taxonomy flow chart will showcase the complex relationships between various theories in different research domains [45]. (3) A summary table is crafted to encapsulate the characteristics of each study. (4) A summary table for opportunities and challenges will systematically lay out the inherent advantages and disadvantages tied to the methods identified [46].

Considering the implications discussion, we aim to contextualize our results by aligning the research purpose with the research findings. This reflection will pave the way for potential future research directions, policy formulations, and practical applications. The insights of this discussion will be interesting for a diverse set of stakeholders, from academicians to industry professionals and researchers who want to explore the cognitive process of test takers.

### Consultation

Consultation is an optional stage for conducting a scoping review. The purpose of this stage is to ask stakeholders to offer additional information, support, and contribution. Our study aims to explore an emerging research area related to log file data, so we will incorporate content experts for knowledge transfer and exchange. Experts with experience in related fields, such as psychometrics, education measurement, and computer science, will be invited to consult their suggestions and criticism about this scoping review, especially for exploring the needs and interests of the log file data from their practice.

## Discussion

Log file data has emerged as a significant research topic in the fields of psychology and education. The widespread adoption of computer-based assessment has made log file data increasingly accessible. However, the substantial increase in volume, velocity, and variety of log file data raise new challenges for researchers in terms of management, analysis, and interpretation to fully realize its potential [1]. A notable gap exists in the form of a comprehensive scoping review to provide a comprehensive overview of the current theoretical and analytical frameworks to guide future research and practice, which is essential to guide future research and practice.

The proposed scoping review aims to identify, classify, and synthesize the evidence on the application of log file data, specifically addressing the inherent opportunities and challenges. This protocol delineates a comprehensive plan for conducting the review, which includes outlining the theoretical framework, detailing the study design, conducting database searches, selecting relevant studies, and projecting potential outcomes. Publishing this protocol is intended to not only enhance the reproducibility of the study but also to be benefit from the feedback of experts in the field.

## Proposed timeline and work allocation

Additionally, this scoping review is anticipated that it will be completed within 8 months, the proposed timeline and work allocation are outlined in Table 2 below.

**Table 2:** Proposed timeline and work allocation for the scoping review.

Stage	Task	Time	Work Allocation
1	Identifying research questions and writing the protocol	1 month	GC, YL
2	Refining search strategy and updating inclusion criteria	1 month	GC, YL
3	Study selection	1 month	GC, YM
4	Data extraction	2 months	GC, YM
5	Data Synthesis	2 months	GC, Content experts
6	Consultation and writing the manuscript	2 months	GC, YL

## Supporting information

S1 Checklist Preferred Reporting Items for Systematic reviews and Meta-Analyses extension for Scoping Reviews (PRISMA-ScR) checklist. (DOCX)

S1 File (PDF)

S2 File (PDF)
