# Supplementary material for: Understanding the log file data from educational and psychological computer-based testing: A scoping review protocol
Source: PLoS One. 2024 May 23;19(5):e0304109. doi: 10.1371/journal.pone.0304109 (PMC11115232; doi:10.1371/journal.pone.0304109)
Supplement: S1 File — (PDF) [file pone.0304109.s002.pdf]

S2 Details of search query

Blocks	Fields	Explanation
"process data" OR “log data” OR “log file” OR logfile)	Title, abstract, and keywords	Incorporates all terms associated with log file data.
computer based test OR computerized test OR assessment OR “scenario based” OR PISA OR PIAAC OR NAEP OR TIMSS OR PIRLS	Title, abstract, and keywords	Incorporates all terms associated with computer-based testing.
2000-2023	Publication Year	Restricts the publication year range to 2000-2023.
English	Refine the retrieved results	Narrows the search to documents published in English.

Note. The aim is to construct our search query with as broad a scope as possible. Quotations are employed to ensure the exact phrase is retrieved. Modern databases will automatically address the singular and plural (https://images.webofknowledge.com/WOKRS521R5/help/WOK/hs_topic.html) and hyphenated and non-hyphenated form (https://images.webofknowledge.com/images/help/WOS/hs_search_rules.html#:~:text=If%20you%20enter%20two%20words,or%20the%20phrase%20waste%20water.)

Database	Query	Notes
Web of Science	(TS = (("process data" OR “log data” OR log-file OR logfiles) AND (computer based test OR computerized test OR "scenario based" Or assessment OR PISA OR PIAAC OR NAEP OR TIMSS OR PIRLS)) AND PY = (2000-2023))	1546 papers were found. Searching by Topic terms includes Title, Abstract, and Author Keywords.
ERIC, Education Source, PsycInfo (EBSCOhost)	TI ((("process data" OR “log data” OR “log file” OR logfile) AND (computer based test OR computerized test OR assessment OR “scenario based” OR PISA OR PIAAC OR NAEP OR TIMSS OR PIRLS))) OR AB ((("process data" OR “log data” OR “log file” OR logfile) AND (computer based test OR computerized test OR assessment OR “scenario based” OR PISA OR PIAAC OR NAEP OR TIMSS OR PIRLS))) OR SU ((("process data" OR “log data” OR “log file” OR logfile) AND (computer based test OR computerized test OR assessment OR “scenario based” OR PISA OR PIAAC OR NAEP OR TIMSS OR PIRLS))	565 papers were found.
ProQuest Dissertations & Theses Global	title((("process data" OR "log data" OR "log file" OR logfile) AND	173 papers were found.

	(computer based test OR computerized test OR assessment OR "scenario based" OR PISA OR PIAAC OR NAEP OR TIMSS OR PIRLS))) OR abstract((("process data" OR "log data" OR "log file" OR logfile) AND (computer based test OR computerized test OR assessment OR "scenario based" OR PISA OR PIAAC OR NAEP OR TIMSS OR PIRLS))) OR subject((("process data" OR "log data" OR "log file" OR logfile) AND (computer based test OR computerized test OR assessment OR "scenario based" OR PISA OR PIAAC OR NAEP OR TIMSS OR PIRLS)))	
Scopus	TITLE-ABS-KEY ((("process data" OR "log data" OR "log file" OR logfile) AND (computer AND based AND test OR computerized AND test OR assessment OR "scenario based" OR pisa OR piaac OR naep OR timss OR pirls))) AND PUBYEAR > 1999 AND PUBYEAR < 2024 AND (LIMIT-TO (LANGUAGE , "english"))	434 papers were found.

Note: Search conducted on February 9th, 2024. Due to database-specific limitations, researchers may need to manually apply language filters. A total of 2146 papers were identified after removing duplicates using Covidence.
