# Supplementary material for: Understanding the log file data from educational and psychological computer-based testing: A scoping review protocol
Source: PLoS One. 2024 May 23;19(5):e0304109. doi: 10.1371/journal.pone.0304109 (PMC11115232; doi:10.1371/journal.pone.0304109)
Supplement: S2 File — (PDF) [file pone.0304109.s003.pdf]

S3 Data extraction form

Attached is the printed Google Form for data extraction for *Understanding the Log File Data from Educational and Psychological Computer-Based Testing: A Scoping Review Protocol*.

Log File Data

This is the data extraction form for log file data.

* Indicates required question

Part 1: Study Information

This part only records the basic study information

1. ID *

   The ID from the Covidence.

2. Study Title *

3. Lead Author Name *

4. Publication Year *

   Year only

5. Journal or Conference Proceedings Name *

Part 2: Study Summary

6. Study Purpose *

   if it is not provided, you may summarize it by yourself.

7. Research Questions *

8. Study Design *

Mark only one oval.

- Observational study
- Quasi-experimental study
- Experimental study

9. Conclusions and Findings *

10. Opporutnity: part one *

Did the author mention the opportunities of applying the logfile data?

Mark only one oval.

- Yes
- No
- Other:

11. Opporutnity: part two

If yes, please summarize the opportunties provided.

12. Challenge: part one *

Did the author mention the challenges of using logfile data?

Mark only one oval.

- Yes
- No
- Other:

13. Challenge: part two

If yes, please summarize the challenges provided.

Part 2: Study Characteristics

If one paper include more than one empirial studies, please fill in another new form.

14. Data Source *

Mark only one oval.

- ( ) PISA
- ( ) PIAAC
- ( ) NAEP
- ( ) Other: ______________________

15. Year of the Data Collection *

If the data is from a large-scale assessment (i.e., PISA, PIAAC and so on), please indicate the year of dataset.

____

16. Subject *

Mark only one oval.

- ( ) Reading
- ( ) Mathematics
- ( ) Problem-solving
- ( ) Other: ______________________

17. Sample Size *

____

18. Methods and software for data cleaning *

____

19. Methods and software for feature extraction *

____

Part 3: Analytical Methods

20. Method Name *

What is the specific name of the method?

____

21. Type of Method *

Mark only one oval.

- ( ) Decriptive statistics
- ( ) Inferential statistics
- ( ) Machine learning: supervised learning *Skip to question 22*
- ( ) Machine learning: unsupervised learning *Skip to question 22*
- ( ) Other: ______

Skip to question 25

Part 3.1: Analytical Framework

If machine learning approaches are used, this part need to be filled out.

22. Performace Metrics *

Check all that apply.

- [ ] F1
- [ ] AUC
- [ ] Other: ______

23. Cross-Validation *

Mark only one oval.

- ( ) K-fold
- ( ) Leave-p-out
- ( ) Nested
- ( ) Other: ______

24. Cognitive and Psychological Variable *

Is there any cognitive and psychological variable?

Mark only one oval.

- ( ) Yes *Skip to question 25*
- ( ) No
- ( ) Other: ______

Part 3.2: Theoretical Framework

If the study includes any psychological variable, please provide details about the underlying theory.

25. Theoretical Model *

Did authors adopt any psychological or educational theoritical model?

Mark only one oval.

- Yes
- No
- Other: ________________

26. Theoretical Model

Specify the theoretical model

____

27. Reference for the Theoretical Model

Please find the reference for this theoretical model

____

This content is neither created nor endorsed by Google.

Google Forms
